# HIV test: which is your best? A National survey on testing preferences among MSM in Italy

**DOI:** 10.7448/IAS.17.4.19598

**Published:** 2014-11-02

**Authors:** Sandro Mattioli, Giulio Maria Corbelli, Stefano Pieralli, Michele Degli Esposti

**Affiliations:** 1 Plus onlus, na, Bologna, Italy; 2 European AIDS Treatment Group, na, Bruxelles, Belgium

## Abstract

**Introduction:**

HIV testing opportunities in Italy are frequently limited to the hospital setting. Experiences in other countries show that offering HIV testing in other facilities could improve HIV testing uptake.

**Methods:**

An internet-based survey was conducted between March 10 and April 3, 2014.

**Results:**

A total number of 348 questionnaires were collected. Responders were 88% male. Most represented age groups were 25–34 (35%) and 35–44 (25%). Most of the responders identify themselves as homosexual (81%) or bisexual (9%). Half of responders had an HIV test within 2 years (56%) while 18% never tested for HIV. Among all responders, 61% had more than 2 sexual partners in the past year. Reported condom use in the past year was: always 39%, always but once 11%, sometimes 27%, never 14%. Most known places to have an HIV test is the hospital (95%), STI clinic (58%) and chemical analysis laboratory (54%); most used places are hospital (73%), STI clinic (30%), laboratory (22%) while 5 responders reported having had a self-test at home. Preferred places where to have an HIV test is self-testing at home (53%), hospital (36%), pharmacy (32%) and headquarter of an organization (31%). Most known testing method is draw blood from vein (97%), which is also most used (80%) but the least preferred (31%) while saliva (65%) and finger prick (56%) are the preferred choices. Most responders know that physicians (84%) and nurses (77%) are those who perform HIV tests and most of them had an HIV test with them (60% and 65% respectively). Physicians are the preferred operators (54%) followed by self-testing (46%), nurses (46%) and peer-volunteers (39%). The ideal HIV test should be: reliable (86%), with no medical prescription (75%), free (63%), rapid (55%), with no personal information collected (45%), with the opportunity to speak with a peer-counsellor (36%).

**Conclusions:**

Changing HIV testing policies in Italy is urgently needed in order to grant a better access to the service: waiting for the results and bureaucratic obligations represent the major barriers to be removed. Home-testing and community-based testing seem to be among the best ways to offer new opportunities though they may require a change in the legal, social and cultural context to be implemented and home testing will not allow any kind of support for newly diagnosed people.

**Table 1:** Survey results

Which places for HIV testing do you know?	%	N	Which places for HIV testing did you use?	%	N	Which places for HIV testing would you like to use?	%	N	Which procedures for HIV testing do you know?	%	N	Which procedures for HIV testing did you use?	%	N	Which procedures for HIV testing would you like to use?	%	N	Which operator for HIV testing do you know?	%	N	Which operator for HIV testing did you use?	%	N	Which operator for HIV testing would you like to use?	%	N	How would your preferred test be?	%	N
Hospital	94.8	330	Hospital	73.0	254	Hospital	36.5	127	Vein blood draw	96.6	336	Vein blood draw	80.5	280	Vein blood draw	31.3	109	Physician	83.9	292	Physician	60.1	209	Physician	54.0	188	It's mandatory to be free	63.2	220
STI clinic	57.8	201	STI clinic	30.5	106	STI clinic	27.3	95	Finger prick	30.5	106	Finger prick	5.5	19	Finger prick	55.7	194	Nurse	76.7	267	Nurse	64.9	226	Nurse	45.7	159	I can pay 5–10 € to have it as I want it	39.4	137
Drug abuse service	23.6	82	Drug abuse service	2.3	8	Drug abuse service	4.6	16	Saliva	43.1	150	Saliva	10.6	37	Saliva	65.2	227	Pharmacist	2.6	9	Pharmacist	0.3	1	Pharmacist	20.7	72	Medical prescription must not be necessary	74.7	260
Pharmacy	2.6	9	Pharmacy	0.3	1	Pharmacy	31.9	111	Don't know	0.6	2	Never tested	16.7	58	HIV+, will not test	16.1	56	Lab operator	39.9	139	Lab operator	19.3	67	Lab operator	27.6	96	Result must be available rapidly (20–30 minutes)	55.7	194
Analysis Lab	53.7	187	Analysis Lab	21.6	75	Analysis Lab	20.7	72				Don't know	0.3	1	Don't know	0.6	2	Volunteer	23.9	83	Volunteer	9.8	34	Volunteer	38.5	134	It's mandatory to be extremely reliable	85.9	299
Street unit	25.9	90	Street unit	3.2	11	Street unit	22.4	78										Myself (home-testing)	14.4	50	Myself (home-testing)	2.3	8	Myself (home-testing)	46.0	160	Name, surname, ID must not be requested	44.5	155
Org. Headquarter	23.6	82	Org. Headquarter	6.9	24	Org. Headquarter	31.0	108										Don't know	1.1	4	Never tested	16.4	57	HIV+, will not test	14.9	52	I want a physician to be present	21.0	73
Home testing	8.6	30	Home testing	1.4	5	Home testing	52.6	183													Don't know	0.3	1	Don't know	1.4	5	Don't want to receive counselling	29.0	101
Don't know	0.9	3	Never tested	16.1	56	HIV+, will not test	10.6	37																			I want to receive peer-counselling	35.6	124
			Don't know	0.6	2	Don't know	1.1	4																			I want to receive counselling from a physician	23.9	83
																											Other	0.9	3

